# The Role of Decorin in Autoimmune and Inflammatory Diseases

**DOI:** 10.1155/2022/1283383

**Published:** 2022-08-17

**Authors:** Yuanji Dong, Jixin Zhong, Lingli Dong

**Affiliations:** Department of Rheumatology and Immunology, Tongji Hospital, Tongji Medical College, Huazhong University of Science and Technology, Wuhan, Hubei, China

## Abstract

Decorin is an extracellular matrix protein that belongs to the family of small leucine-rich proteoglycans. As a matrix protein, the first discovered role of decorin is participating in collagen fibril formation. Many other functions of decorin in various biological processes have been subsequently identified. Decorin is involved in an extensive signaling network and can interact with other extracellular matrix components, growth factors, receptor tyrosine kinases, and various proteases. Decorin has been shown to be involved in wound repair, cell cycle, angiogenesis, tumor metastasis, and autophagy. Recent evidence indicates that it also plays a role in immune regulation and inflammatory diseases. This review summarizes the characteristics of decorin in immune and inflammatory diseases, including inflammatory bowel disease (IBD), Sjögren's syndrome (SS), chronic obstructive pulmonary disease (COPD), IgA nephropathy, rheumatoid arthritis (RA), spondyloarthritis (SpA), osteoarthritis, multiple sclerosis (MS), idiopathic inflammatory myopathies (IIM), and systemic sclerosis (SSc) and discusses the potential role in these disorders.

## 1. Introduction

Autoimmune and inflammatory diseases are major health problems affecting over 200 million people worldwide [1]. The search for new therapeutic approaches is not only to help understand the process of disease occurrence and development but also to alleviate patients' symptoms and reduce the economic burden on society. The extracellular matrix is a well-organized complex collection of different proteins, including collagen, elastin, proteoglycan, and glycosaminoglycan [2]. Proteoglycans, which consist of one or more sulfated glycosaminoglycan chains and core proteins, include large proteoglycans and small leucine-rich proteoglycans (SLRPs) [3, 4]. SLRPs can be divided into five distinct classes based on their number of LRRs, amino acid residues at the N-terminus, and their chromosomal organization. Decorin belongs to the first class of SLRPs. In humans, the core protein of decorin is made up of 10-12 repeating leucine-rich motifs, and the GAG chain covalently attaches to the amino terminus via a serine residue [5]. The GAG side of decorin is usually dermal sulfate (DS) or chondroitin sulfate (CS), depending on the tissue. Skin, tendons, and intima arteriae are mainly DS, while bone and cartilage are mainly CS [6]. Decorin has multiple functions and is not only localized to the extracellular matrix and dense connective tissues such as tendons and ligaments but also exists in the body fluid, including plasma and aqueous humor [7–13]. In addition to interacting with collagen, soluble decorin is also involved in various biological processes, including inflammation, autophagy, angiogenesis, cell cycle, wound healing, and fibrosis [14–20]. Previous studies focused on its inhibitory effect on fibrosis and tumor. As an endogenous antagonist of transforming growth factor *β* (TGF-*β*), decorin can physically interact with TGF-*β*, interfere with TGF-*β* signaling, and form decorin/TGF-*β* complexes in the extracellular matrix, thereby significantly attenuating the profibrotic effect of TGF-*β* [21]. In tumors, decorin has been shown to inhibit metastasis, tumor cell proliferation, and angiogenesis and regulate autophagy and inflammation [22]. In addition to these characteristics, decorin, as one of the damage-associated molecular patterns (DAMPs), can initiate aseptic inflammation and induce the activation of innate immune cells, which provides the basis for its involvement in autoimmune and inflammatory diseases [11, 23]. This review article summarizes the characteristics of decorin in immune and inflammatory diseases and discusses the potential role of decorin in autoimmune diseases.

### 1.1. The Structural Characteristics of Decorin

**Figure 1:**
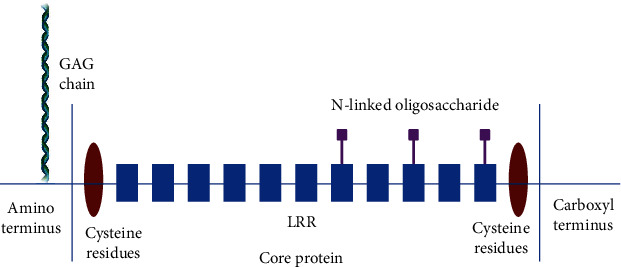
The structure of decorin. Decorin consists of amino-terminal domains, glycosaminoglycan chains, core protein domains, and carboxy-terminal domains. The binding site of the GAG (glycosaminoglycan) chain exists in the amino-terminal domain. The core protein domain contains leucine repeats (LRR) and N-linked-oligosaccharides. The remainder is the carboxy-terminal domain.

Decorin, also known as PG40, is a member of the small leucine-rich proteoglycan (SLRPs) family [24, 25]. Mature decorin contains a 42 kDa protein core with a sulfated glycosaminoglycan chain attached to its N-terminal [24]. The protein core contains twelve leucine-rich repeats (LRR) flanked by a cysteine-rich region [26, 27] (Figure 1). The decorin has a horseshoe-shaped appearance, with fourteen *β*-sheets at the concave surface and many *α*-helices at the convex surface [26, 28]. In physiologic conditions, decorin exists as a dimer; however, this process is reversible, which is mediated by the concave surfaces of decorin, and the dimerization of decorin prevents its core region from binding to other substrates, implying that monomeric decorin accounts for most of the interactions [29, 30]. Furthermore, decorin can generate complex context-dependent interactions with many ligands through GAG chains and core proteins [24]. Despite its complex function, decorin knockout mice were fertile and viable. No significant morphological abnormalities were observed, except reduced skin and tendon mechanics, suggesting a role of decorin in collagen fiber formation [6]. On the other hand, the study also found that the function of decorin overlaps with other SLRPs such as biglycan and asporin, which relieves the symptoms after decorin knockout [31, 32].

### 1.2. The Source and Expression of Decorin

Decorin is mainly expressed in fibrous connective tissue and primarily involves collagen fiber formation in the dermis, cornea, tendons, and cartilage. Decorin is typically synthesized and secreted by fibroblasts, maintaining the dynamic balance of the extracellular matrix. However, epithelial cells and endothelial cells can also synthesize decorin; although, they do not constitutively express it [9]. Many factors can modulate the expression of decorin. Among them, the effect of inflammation and cytokines on the decorin expression is interesting. Decorin is significantly induced in inflammation-related angiogenesis in vivo but not in noninflammation-dependent angiogenesis. However, inflammatory cytokines failed to directly induce decorin synthesis in endothelial cells, suggesting that decorin may act on endothelium through paracrine processes [9, 33]. Recent studies have also shown that decorin can be released early after ferroptosis and participate in the proinflammatory responses [34]. Cytokines interleukin-6 (IL-6) and IL-10 have been shown to upregulate the expression of decorin in smooth muscle cells and endothelial cells, whereas tumor necrosis factor *α* (TNF-*α*) and TGF-*β* inhibit the transcription of decorin [35–37]. The effect of IL-1 and IL-4 on the expression of decorin is controversial. Both increasing and inhibiting effects on the transcription have been reported [35, 38–40]. In addition, several studies have reported that immune cells are able to synthesize and secrete decorin. Lipopolysaccharide (LPS) stimulation increased the decorin transcription and secretion levels in peritoneal macrophages [11]. In asthmatic patients and mouse models, both CD4+ T and CD8+ T cells showed an upregulated expression of decorin [41].

### 1.3. Signaling Network Initiated by Decorin

Decorin has been linked to several biological functions. It can directly participate in collagen formation, act as a ligand to bind relevant receptors to mediate downstream signal transduction, and block specific cytokines and growth factors to inhibit downstream signal transduction. Decorin binds to various collagen fibers (including I, II, III, IV, V, VI, XII, and XIV) which type 1 collagen is the most widely studied [30, 42]. It has been demonstrated that the triple helix of type 1 collagen has a site in both “*D*” and “*E*” bands that can bind to the core protein of decorin. Such a structure can prevent abnormal fusion between collagen molecules [26]. The core protein of decorin binds to collagen fibrils at a uniform spacing of 65 nm, and the charged GAG chain is perpendicular to the collagen fibrils and connects adjacent fibrils, regulating the distance between fibrils. Furthermore, the GAG chain may attach to tenascin-X, modulating its effects on collagen and ECM integrity, and decorin can acts as a bridging molecule binding different collagens [5]. These are important for the accurate arrangement and localization of collagen fibrils in the ECM. So, the lack of decorin leads to variations in the diameter of fibril, and the spacing and biomechanics are impaired. In addition, decorin can interact with other extracellular matrix components such as matrilin-1, tenascin X, microfilament-associated protein (MFAP-2), and fibrillins, which are involved in tissue cell adhesion and migration to maintain the mechanical strength of connective tissue [43–45].

**Figure 2:**
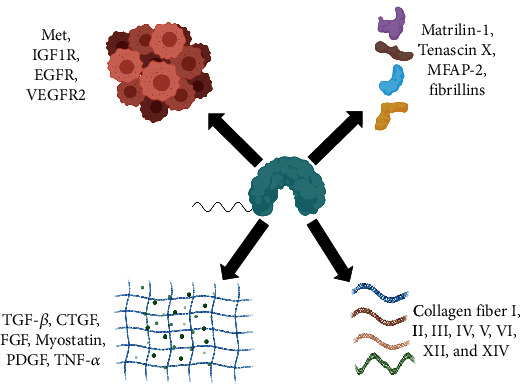
The biological signaling network of decorin. Decorin binds to collagen fibers (including I, II, III, IV, V, VI, XII, and XIV). In addition, decorin can interact with other extracellular matrix components such as matrilin-1, tenascin X, MFAP-2, and fibrillins. Decorin can bind to receptors on the surface of tumor cells (such as Met, IGF1R, EGFR, or VEGFR2). Moreover, decorin also acts as a reservoir for cytokines (TGF-*β*, myostatin, CTGF, FGF, PDGF, and TNF-*α*).

Decorin can also act directly on receptors on the cell surface. Met, encoded by protooncogene c-Met, is a tyrosine kinase receptor that plays an essential role in cell migration, apoptosis, proliferation, and differentiation. Studies showed that decorin could bind to Met on the surface of tumor cells, leading to rapid receptor phosphorylation and degradation in the endosomes, and could also induce mitochondrial autophagy by activating Met [46–48]. Decorin could also target insulin-like growth factor 1 receptor (IGF1R) on the surface of cancer cells and inhibit its downstream signaling or target vascular endothelial growth factor receptor 2 (VEGFR2) on the surface of endothelial cells to promote autophagy [49, 50]. Decorin can also inhibit tumor growth by blocking epidermal growth factor receptor (EGFR) and ErbB4 dimerization [51]. Decorin could also serve as a reservoir for TGF-*β*, myostatin, connective tissue growth factor (CTGF), fibroblast growth factor (FGF), platelet-derived growth factor (PDGF), and TNF-*α* to maintain their homeostasis, which requires complex feedback mechanisms and strict regulatory networks [30] (Figure 2).

### 1.4. Decorin and Inflammation

Decorin, as endogenous ligands, binds to TLR2 and TLR4 on the surface of macrophages with an affinity comparable to that of pathogen ligands, triggering an acute inflammatory response, leading to rapid activation of P38, ERK1/2, and NF-*κ*B pathways and the synthesis of proinflammatory factors TNF-*α* and IL-12p70 [52]. In addition, by signaling through TLR2/4, decorin also acts as a transcriptional inducer of the tumor suppressor programmed cell death 4 (PDCD4), a specific translational suppressor of IL-10, maintaining a proinflammatory environment [52]. However, the core protein alone may play a role in inhibiting inflammation in a triple-negative orthotopic breast cancer xenograft model by competitive inhibition of other DAMP molecules bound to TLR2 and TLR4 [53]. In addition to immune cells, Toll-like receptors were also expressed in some tissue cells, such as annulus fibrosus cells and salivary gland epithelial cells, which both expressed TLR4 [54, 55]. The former can produce MIP-2 in response to decorin stimulation, and the latter can increase TNF-*α* transcription level in response to decorin stimulation, and both of them can be inhibited by TAK-242 (TLR4 inhibitor). In addition, decorin also plays a proinflammatory role in several diseases. In chronic pancreatitis, decorin significantly increases macrophage inflammatory protein-1 (MCP-1) level in peripheral blood mononuclear cells [56]. In delayed hypersensitivity models, decorin enhances interferon-*γ* (IFN-*γ*) signal transduction by activating signal transduction transcriptional activator 1 (STAT-1) [14].

It is worth noting that only the intact decorin composed of core protein and the glycosaminoglycan (GAG) chain can trigger the proinflammatory signal. The intact decorin is sensitive to various enzymes, including matrix metalloproteinases- (MMP-) 2, 3, 7, and 8 [16, 57, 58], which can digest and destroy core proteins, destabilize the matrix, and release cytokines (TGF-*β* or TNF-*α*) previously bound to decorin. Furthermore, the regulation of decorin in the inflammatory response is complicated and depends on the context of inflammation. In ischemia-reperfusion injury, TGF-*β*1 is involved in the aggravation of tissue damage after perfusion, and decorin has a potentially protective effect by inactivating TGF-*β* [59]. In addition, intraperitoneal injection of decorin after traumatic brain injury significantly reduced caspase 3 activity, increased superoxide dismutase levels, and protected brain tissue and neurons [60].

### 1.5. Decorin and Autophagy

Autophagy is a self-degrading process that degrades damaged organelles and misfolded and aggregated proteins through the lysosomal pathway [61]. Physiological autophagy is essential for normal cell function, signal transduction, and proliferation, but pathological autophagy is involved in various diseases, including autoimmune diseases [62]. Decorin is the first extracellular matrix component identified to influence cellular metabolic processes. The role of decorin in mitochondrial autophagy in epithelial-derived tumors and endothelial cells has been widely reported [22]. Decorin induced the expression of mitostatin in breast cancer, leading to mitochondrial ultrastructural changes [63, 64]. Decorin could also trigger mitochondrial depolarization and cause the translocation of Parkin from the cytoplasm to mitochondria, leading to the ubiquitination of mitochondrial proteins [65, 66]. Decorin stimulated polyethylene glycol 3 (PEG3) synthesis in endothelial cells by interacting with VEGFR2. Decorin could also inhibit the upregulation of mTOR after VEGFA binding to VEGFR2, which resulted in enhanced TFEB expression and nuclear translocation, promoting autophagosome formation [50, 67, 68]. Decorin has recently been suggested to induce autophagy in intestinal inflammatory epithelial cells [69]. Therefore, decorin may induce autophagy in both cancer and inflammatory conditions, which, however, may need further studies to confirm.

### 1.6. The Interaction between Decorin and Immune Cell

**Figure 3:**
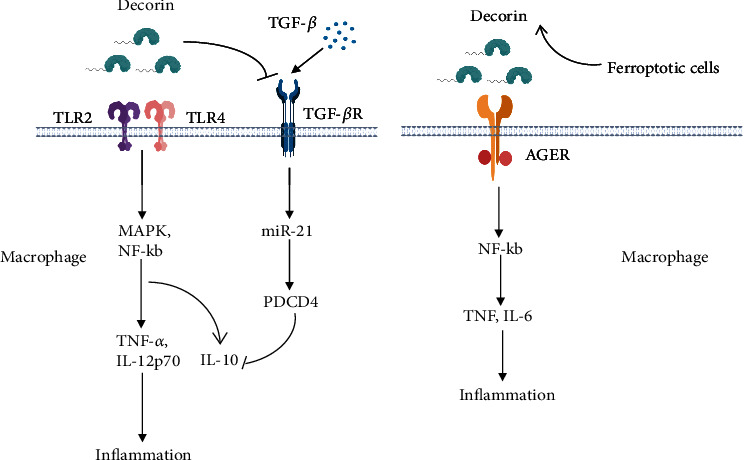
The role of decorin on macrophages. Soluble and intact decorin can act on TLR receptors (TLR2 and TLR4) on the surface of macrophages, activating downstream MAPK and NF-*κ*B pathways, resulting in increased cytokines (TNF-*α*, IL-12P70, and IL-10). Furthermore, decorin antagonizes the effects of TGF-*β*, leading to a decrease in IL-10, which maintains the proinflammatory function. On the other hand, decorin released by ferroptosis can also act on AGER receptors on macrophages, leading to downstream NF-*κ*B pathway activation and increased TNF and IL-6.

Decorin can theoretically interact with multiple immune cell types because TLR is expressed on many immune cells. However, the current research in this area is still lacking, with most of them focusing on macrophages. Autocrine- and paracrine-released decorin stimulated TLR2 and TLR4 receptors on macrophage surface, activated inflammatory pathways of P38, MAPK, and NF-*κ*B, and enhanced the production of proinflammatory factors (IL-12p70 and TNF-*α*) [11, 23, 70]. However, decorin can activate other receptors as well. Recent studies showed that extracellular decorin bound to advanced glycosylation end product-specific receptor (AGER) on macrophages triggers proinflammatory cytokine production in an NF-*κ*B dependent manner [34] (Figure 3). Decorin also indirectly affected the foxp3 gene expression through the TGF-*β* signaling pathway [71]. In DCN-/- mice, the biological activity of TGF-*β*, CD4+CD25+Foxp3+ T lymphocytes, and IL-10 levels were increased, which inhibited the Th2 cell immune response in allergic asthmatic mice [71].

## 2. Decorin in Autoimmune and Inflammatory Diseases

Decorin is a multifunctional protein that plays a vital role in various biological processes. Decorin may be involved in immune-related diseases for several important reasons. First, as one of the DAMPs, it can activate pattern recognition receptors such as TLR on innate immune cells. Secondly, as an endogenous TGF-*β* blocker, it reduces the level of anti-inflammatory factor IL-10 and maintains the persistence of inflammation. Finally, it can induce autoantibodies against decorin; although, its significance has not been elucidated [70]. Previous studies on autoimmune and inflammatory diseases have focused on the interactions between immune cells and immune cells and between immune cells and parenchymal tissue cells. Still, there has been limited research on the interaction between immune cells and extracellular matrix. The extracellular matrix is one of the most abundant tissue components in the body. Although its role in disease has not been fully elucidated, it has significant therapeutic potential. This review summarizes the role of decorin in diseases including IBD, SS, COPD, IgA nephropathy, MS, IIM, RA, and osteoarthritis (Table 1).

### 2.1. IBD

Inflammatory bowel disease (IBD) is a nonspecific chronic inflammatory disease, including Crohn's disease (Th1 dominant immune response) and ulcerative colitis (Th2 dominant immune response) [72]. The role of decorin in IBD has been explored only in mouse models and in vitro cell lines. The study found increased levels of decorin and autophagy-related proteins Beclin1 and LC3b in the intestinal wall of IBD mice. The overexpression of decorin in human colon epithelial cells resulted in increased autophagosomes and decreased apoptosis, suggesting that decorin may play a protective role in inflammatory bowel disease by increasing the autophagy of epithelial cells and decreasing apoptosis [69]. However, further research is needed to clarify the relationship between decorin and inflammation disease, for example, whether there is a clear interaction between decorin and the immune cells or is there a long-term effect of epithelial autophagy and whether this could lead to dysfunction of epithelial cells.

### 2.2. SS

Sjögren's syndrome (SS) is a common autoimmune disease involving exocrine glands (salivary and lacrimal). About 40% of patients develop exocrine symptoms, including muscle arthralgia, interstitial lung disease, and central nervous system involvement, and about 5% develop lymphoma [73]. The roles of epithelial cells and immune cells in Sjögren's syndrome have been extensively studied. However, the treatment selection of Sjögren's syndrome is still limited [74–76]. The evidence of the involvement of decorin in SS is growing. An earlier study found an increased MMP activity and an enhanced decorin degradation in the exocrine glands of NOD mice. However, given the limited activities of cleaved decorin, the role of decorin in SS is not precisely clarified [77]. In a primary Sjögren's syndrome mouse model (NOD.B10), decorin was found to induce TNF-*α* production and reduce MIP-1*α* and MCP-1 in the spleen via TLR4 rather than TLR2 [78]. This indicates that decorin may have a different effect on different cytokines/chemokines and immune cells in SS. Further studies showed that although circulating levels of decorin were not different between NOD.B10 mice and control mice, autoantibodies against decorin were significantly elevated in pSS mice, and decorin was highly expressed in the salivary gland, lung, and kidney tissues of pSS mice [79]. Our study also found that decorin was significantly elevated in the salivary glands in the experimental Sjögren's syndrome model and pSS patients, and decorin induced the apoptosis of A253 cells and polarization of macrophages towards the M1 phenotype [54]. Current evidence supports the ECM degradation products as a new source of B cell activation in SS. However, more evidence is needed to clarify whether decorin can be used as an early therapeutic target in SS.

### 2.3. COPD

Chronic obstructive pulmonary disease (COPD) is a chronic airway disease with restricted airflow. There is increasing evidence that COPD is associated with immune abnormalities, and some patients present with autoimmune reactions [80]. These abnormalities are characterized by the formation of B cell lymphoid follicles in the lung tissue and the presence of anti-HEP-2 epithelial cells and antielastin and antidecorin autoantibodies in the serum of patients with COPD [81–85]. The level of decorin secreted by fibroblasts from patients with severe COPD was decreased, and the expression of decorin was also reduced in pulmonary mesenchymal stem cells (LMSCs) from COPD patients [86]. More antidecorin IgG was produced when PBMCs from COPD patients were stimulated by a combination of extracellular matrix components and cytokines [85]. However, when the mice were immunized with extracellular matrix and exposed to cigarette smoke, an immune response specific to decorin was induced; although, it did not enhance smoke-induced inflammation. However, it cannot be ruled out that autoantibodies against decorin may cause tissue damage over a longer time [87]. In addition, decorin in the peripheral blood of patients with COPD has recently been reported as a predictor of acute disease exacerbation [88]. The current evidence supports that decorin has some immunogenicity. However, more evidence is needed to support the involvement of decorin in COPD.

### 2.4. IgA Nephropathy

IgA nephropathy is a common primary glomerular disease caused by the deposition of immune complexes in the mesangial region, resulting in mesangial cell proliferation [89]. Decorin is mainly secreted by renal fibroblasts in the normal kidneys and located in the renal tubule interstitium [90]. In IgA nephropathy, the transcriptional level of decorin was increased and was mainly located in sclerotic glomeruli and fibrotic sites. These results suggest that decorin is involved in the pathogenesis of IgA nephropathy [91]. A recent study investigated the effects of TGF-*β*1 and decorin on podocyte autophagy. The results showed that TGF-*β*1 could activate the mammalian target of rapamycin complex 1 (mTORC1) to inhibit podocyte autophagy and participate in podocyte apoptosis [92]. In addition, this study also found that podocytes may be a source of decorin [92]. However, it is exciting and necessary further to investigate the mechanism of decorin in IgA nephropathy.

### 2.5. RA, SpA, and Osteoarthritis

Rheumatoid arthritis (RA) is characterized by synovial hyperplasia, pannus formation, and bone/cartilage damage [93]. Lymphoid follicles and ectopic germinal centers were found in the synovial tissues, suggesting that the cartilage matrix may be a potential component of autoantigens in RA [94]. It was reported that the frequency of IgM antibodies against decorin was the highest among all matrix molecules. Decorin binds C1q to inhibit the classical pathway of complement under normal conditions, while autoantibodies bind decorin and activate the classical pathway of complement, which has a potential proinflammatory effect [95]. In addition, decorin normally inhibits TGF*β*, and autoantibodies may affect this process, but the effect on the disease phenotype is complex and unknown [96]. Seronegative spondyloarthropathies (SpA) are chronic inflammatory diseases involving the spine, peripheral joints, ligaments, and tendons [97]. Autoantibodies against decorin were significantly higher in SpA synovial fluid than in OA patients, suggesting that matrix proteins are involved in the chronic inflammatory environment of local joints [96]. Osteoarthritis is a chronic degenerative cartilage disease characterized by joint pain and stiffness [98]. The role of decorin in osteoarthritis is complex. It was reported that serum decorin levels were elevated in OA and could be a risk factor for OA [99]. However, studies in mouse models showed different results. Li et al. found that decorin had a protective effect on cartilage regeneration in posttraumatic osteoarthritis by regulating the fibrogenesis of the cartilage surface [100]. Another study found that the articular cartilage matrix showed higher stiffness and resistance to OA after decorin deletion [101]. These also indirectly illustrate the complexity of decorin in disease, and more studies are still needed.

### 2.6. MS

Multiple sclerosis (MS) is a chronic inflammatory demyelinating disease related to immune dysfunction, often accompanied by sensory, motor, and visual impairment [102]. Decorin in MS is rarely studied, and its function is complex, but it is believed that it has some protection function. In part because it inhibits TGF-*β*, and in the EAE model, inhibition of TGF-*β* signaling may have benefits in the treatment of the acute phase [103]. On the other hand, it is involved in the formation of perivascular fibrosis, which is a typical feature of chronic lesions and can limit the recruitment of immune cells and the expansion of MS lesions [104, 105]. However, more evidence is needed to support whether it can be used as a therapeutic target.

### 2.7. Other Diseases

Idiopathic inflammatory myopathies (IIM) are a heterogeneous group of autoimmune diseases characterized by muscle weakness, inflammatory cell infiltration, and overexpression of MHC1 molecules in muscle fibers [106]. Polymyositis (PM) is mainly infiltrated by CD8+ T cells in the endomysium, while dermatomyositis (DM) is primarily infiltrated by CD4+ T cells in the epimysium. It was reported that decorin could bind and inhibit myostatin from promoting the proliferation and differentiation of myogenic cells [107]. Decorin could also attach to TGF-*β*2 and promote skeletal muscle production [108]. Moreover, the injection of decorin into the injured muscle could induce muscle regeneration [109]. Therefore, decorin can be a potential target in IIM [110].

Systemic sclerosis (SSc) is an autoimmune disease with localized or diffuse skin thickening and fibrosis [111]. Studies have shown that proteoglycan secretion in fibroblasts of SSc patients was significantly increased. Among them, decorin was significantly increased at both transcriptional and protein levels. It is speculated that these changes may affect the composition of the stroma and the course of the disease [112].

## 3. Discussion

Decorin is a versatile protein that interacts with various receptors, enzymes, and cytokines. Decorin is involved in autophagy, cell cycle, inflammation, angiogenesis, and other biological processes. The role of decorin in autoimmune and inflammatory diseases is based on several essential parts. Firstly, decorin, as one of the DAMPs, can participate in the activation of innate immune cells through interacting with TLRs or ARGE receptors. Secondly, the immune system can produce autoantibodies against decorin, which may interfere with the normal function of soluble decorin. Thirdly, decorin is thought to mediate autophagy in endothelial cells or epithelial cells. Finally, decorin can suppress the effects of TGF-*β*, especially in fibrosis. These results suggest that decorin plays a role in developing and progressing autoimmune and inflammatory diseases. Therefore, it is crucial to elucidate the role of decorin in the dynamics of disease development to guide treatment more precisely.

## 4. Conclusion

Decorin has been extensively studied in the process of antifibrosis and antitumor. However, its role in autoimmune and inflammatory diseases is not fully understood. Although several studies have indicated an involvement of decorin in autoimmune and inflammatory disease, the underlying mechanisms remain to be elucidated due to the complexity of decorin in these conditions.

## Figures and Tables

**Table 1:** The role of decorin in autoimmune and inflammatory diseases.

Disease	The role of decorin in disease pathogenesis	References
IBD	The levels of decorin, Beclin1, and LC3b in the intestinal wall of IBD mice were increased; the overexpression of decorin in human colon epithelial cells resulted in increased autophagosomes and decreased apoptosis.	[69]
SS	Degradation products of decorin in the exocrine gland of NOD mice were increased; in a pSS model (NOD.B10), decorin was found to induce TNF-*α* production in spleen tissues via TLR4 and reduce MIP-1*α* and MCP-1 levels in spleen cells; in a pSS model (NOD.B10), autoantibodies against decorin were significantly elevated, and decorin was strongly expressed in salivary gland tissues, lung, and kidney tissues. Decorin was significantly elevated in the salivary glands both in the experimental Sjögren's syndrome model and pSS patients. Decorin induced the apoptosis of A253 cells and polarization of macrophages towards the M1 phenotype.	[54, 77–79]
COPD	The level of decorin secreted by fibroblasts from patients with severe COPD was decreased; using extracellular matrix components and cytokines stimulated PBMC in COPD patients, more antidecorin IgG was produced; immunizing mice with extracellular matrix components induced a specific immune response to decorin; decorin could act as a predictor of acute disease exacerbation in patients with COPD.	[85–88]
IgAN	The transcriptional level of decorin was increased and was mainly located in sclerotic glomeruli and fibrotic sites in IgA nephropathy. Decorin could promote podocyte autophagy and maintain cell homeostasis; podocytes may be a source of decorin.	[91, 92]
RA	The frequency of IgM antibodies against decorin was the highest among all matrix molecules in RA; these antibodies may interfere with the binding of decorin to C1q complement to regulate inflammatory processes.	[96]
SpA	Autoantibodies against decorin were significantly higher in SpA synovial fluid than in OA patients.	[96]
OA	Serum decorin levels were elevated in patients with OA and could be a risk factor for OA; Li et al. found that decorin had a protective effect on cartilage regeneration in posttraumatic osteoarthritis by regulating the fibrogenesis of the cartilage surface; the articular cartilage matrix showed higher stiffness and resistance to OA after decorin deletion.	[99–101]
MS	In perivascular fibrotic tissues of MS, Mohan et al. found the upregulation of decorin, which interacted with fibrillar collagens. Decorin was involved in perivascular fibrosis, which had positive implications for limiting inflammatory cell infiltration and lesion progression.	[104, 105]
IIM	Decorin could bind and inhibit myostatin from promoting the proliferation and differentiation of myogenic cells. Decorin could also attach to TGF-*β*2 and positively affect skeletal muscle production; the injection of decorin into the injured muscle could induce muscle regeneration.	[107–109]
SSc	Decorin was significantly increased at both transcriptional and protein levels in SSc.	[112]

Abbreviation: IBD: inflammatory bowel disease; SS: Sjögren's syndrome; COPD: chronic obstructive pulmonary disease; IgAN: IgA nephropathy; RA: rheumatoid arthritis; SpA: spondyloarthritis; OA: osteoarthritis; MS: multiple sclerosis; IIM: idiopathic inflammatory myopathy; SSc: systemic sclerosis; pSS: primary Sjögren's syndrome; TNF-*α*: tumor necrosis factor *α*; TLR4: Toll-like receptor 4; MIP-1*α*: macrophage inflammatory protein-1 *α*; MCP-1: monocyte chemoattractant protein 1; PBMC: peripheral blood mononuclear cell; TGF-*β*2: transforming growth factor *β*2.

## Data Availability

The data supporting the conclusions of this article will be made available by the authors, without undue reservation.
